# Durable Surface Modification of Low-Density Polyethylene/Nano-Silica Composite Films with Bacterial Antifouling and Liquid-Repelling Properties for Food Hygiene and Safety

**DOI:** 10.3390/polym16020292

**Published:** 2024-01-21

**Authors:** Sang Ha Song, Michael Bae, Jun Kyun Oh

**Affiliations:** 1 Department of Polymer Science and Engineering, Dankook University, 152 Jukjeon-ro, Suji-gu, Yongin-si 16890, Republic of Korea; dkusongsangha@gmail.com; 2 Artie McFerrin Department of Chemical Engineering, Texas A&M University, College Station, TX 77845, USA

**Keywords:** polymer composites, food contact materials (FCMs), superhydrophobic, antifouling, surface treatments

## Abstract

The growing prevalence of antimicrobial resistance in bacterial strains has increased the demand for preventing biological deterioration on the surfaces of films used in applications involving food contact materials (FCMs). Herein, we prepared superhydrophobic film surfaces using a casting process that involved the combination of low-density polyethylene (LDPE) with solutions containing surface energy-reducing silica (SRS). The bacterial antifouling properties of the modified film surfaces were evaluated using *Escherichia coli* O157:H7 and *Staphylococcus epidermidis* via the dip-inoculation technique. The reduction in bacterial populations on the LDPE film embedded with SRS was confirmed to be more than 2 log-units, which equates to over 99%, when compared to the bare LDPE film. Additionally, the modified film demonstrated liquid-repelling properties against food-related contaminants, such as blood, beverages, and sauces. Moreover, the modified film demonstrated enhanced durability and robustness compared to one of the prevalent industry methods, dip-coating. We anticipate that the developed LDPE/nano-silica composite film represents a promising advancement in the multidisciplinary aspects of food hygiene and safety within the food industry, particularly concerning FCMs.

## 1. Introduction

With the expanding world population, there is a growing need for more food, leading to increased demand and consumption of food worldwide. As a result, the management of larger quantities of food products at different stages of the food production process, such as handling, storage, processing, and packaging, has led to a rising global health issue in the form of foodborne illnesses [1]. This has resulted in higher rates of sickness and death [2]. Based on information provided by the World Health Organization, the consumption of contaminated food leads to approximately 600 million instances of foodborne illnesses occurring annually due to the consumption of contaminated food, causing 420,000 fatalities [3]. In the United Kingdom, there are 500,000 reported cases of foodborne illness each year, resulting from recognized pathogens. When accounting for cases triggered by unidentified pathogens, the total count could surpass 1 million [4]. Each year in the United States, around 48 million individuals fall ill, 128,000 require hospitalization, and 3000 die due to foodborne illnesses [5]. There is a growing demand for production that maintains freshness over extended periods and provides protection against both biological and non-biological contaminants [6]. This demand also encompasses the need for antibacterial and antifouling features, which involve cleaning, disinfecting, and sanitizing safeguards [7]. In addressing these requirements, films, an essential category of food contact materials (FCMs), are indispensable components within the materials used in the food industry for applications involving food contact. Innovative food systems can be achieved by integrating nanotechnology with film-based technology. Current research is primarily directed at enhancing the effectiveness of conventional FCMs to improve food quality, safety, and shelf life, and reduce interference with food products [8]. In 2022, the worldwide market for food packaging materials, classified under the term FCMs, surpassed a value of USD 357 billion. It is expected to experience an annual expansion of 6.1% until 2032. This trend is forecasted to result in an estimated total value of USD 642 billion for the market by the year 2032 [9]. Likewise, the global food contact paper market is anticipated to expand from USD 75 billion in 2022 to USD 104 billion by 2029, showcasing a compound annual growth rate (CAGR) of 4.7% throughout the forecast period [10].

To mitigate the occurrence of foodborne illnesses caused by pathogenic bacteria in food contact surfaces, effective strategies are necessary to prevent cross-contamination and the spread of disease-causing bacteria [11]. The majority of food preservation films are manufactured using common polymeric raw materials, such as polyethylene, polypropylene, polyethylene terephthalate, polystyrene, and polybutylene terephthalate [12]. Widely employed in the food industry, these materials play a crucial role in FCMs [13], acting as effective physical barriers against bacterial invasion [14] and contributing to the extension of shelf life [15], thereby maintaining product quality. The surfaces of FCMs acquire a high level of hygiene properties through various mechanisms. One method involves preventing the contamination and proliferation of pathogenic bacteria by coating surfaces with bactericidal agents [16,17]. Bactericidal agents can kill pathogenic bacteria within an effective range and inhibit the formation and growth of bacterial colonies [18]. For example, essential oils from *Rosmarinus officinalis* L. (commonly known as rosemary) [19], *Cyperus rotundus* [20], and *Pistacia lentiscus* [21] exhibit bactericidal properties. Moreover, essential oil from *Atractylodes lancea* has demonstrated antibacterial and antioxidant activities by disrupting cell membranes against bacteria [22]. Another method involves developing bacterial antifouling surfaces by modifying the surface energy, specifically surface hydrophobicity, using a range of coating methods, including thin-film dip-coating, spray deposition coating, continuous blade-coating, and direct roll-coating [23,24]. Modified surfaces have the ability to resist bacterial adhesion [25], reduce bacterial proliferation and infections [26,27], and exhibit self-cleaning properties [28]. As an example, food gloves made from materials such as latex, polyethylene, and nitrile typically have limited resistance to bacterial cross-contamination. However, modification of the surfaces of these gloves with fluorinated nano-silica particles reduces the surface energy, leading to decreased bacterial attachment compared to bare gloves because of the increased hydrophobicity [29]. Currently, while there has been extensive research on FCMs, the majority of these studies have emphasized the biodegradability, antimicrobial capabilities, and antioxidation properties of materials. There are relatively few reports on FCMs designed to prevent fouling and repel contaminants.

The above-mentioned studies including preparation processes such as nanoencapsulation, layer-by-layer deposition, sol-gel synthesis, and thin film coating can indeed be regarded as complex, and their long-term durability and performance persistence may be limited. Therefore, these methods require further consideration for economical and practical applications. The antibacterial properties of films intended for contact with food, which have been modified with bactericidal agents, may not endure permanently. The protective function of material surfaces modified by a coating can easily degrade if deeply scratched, chipped, or cracked by hard, sharp, and heavy objects [30]. If the film coating no longer maintains its resistance to bacterial fouling, it should either be replaced with a new coating or have additional antibacterial agents deposited on it. Bacterial antifouling and repelling is an alternative approach employed in coatings to prevent the attachment of bacterial pathogens and contaminants to surfaces, rather than directly inactivating or killing them. The main drawback associated with these approaches is that, over extended periods of exposure to external wear, the air pockets formed by nanostructures may collapse, displacing the trapped air and consequently diminishing their ability to effectively repel bacteria from surfaces. Also, any imperfections in surface coverage and local flaws within the coating can provide an opportunity for a small number of bacteria to adhere, potentially leading to their reproduction through cell division and the subsequent spreading of bacteria across the surface over an extended period [31]. The durability of the film can be improved using an embedding approach [32]. By reducing the surface energy of polymeric film surfaces by embedding nonpolar hydrophobic agents into the film, the modified surface can suppress the adhesion of substances and maintain longevity compared to the coating approach [33].

In this study, low-density polyethylene (LDPE) film was modified to achieve strong antifouling and repelling properties. To address concerns regarding coating quality degradation, such as short sustainment time, mechanical damage, and chemical leaching from harsh environments (e.g., during ultrasonication, under abrasion, in hydration, within pH), we embedded fluorine-functionalized nanoparticles, referred to as “surface energy-reducing silica (SRS)”, with low surface energy characteristics, into the target film. In accordance with the guidelines provided by the U.S. Food and Drug Administration (FDA) and the European Food Safety Authority (EFSA), silica is considered ‘generally recognized as safe (GRAS)’ as a food additive when orally consumed, with a recommended limit of up to 1500 mg/day [34]. Harmful bacteria associated with foods, including *Escherichia coli*, *Staphylococcus aureus*, *Salmonella enterica*, *Listeria monocytogenes*, and *Bacillus cereus*, are recognized as major contributors to severe foodborne illnesses [35]. Hence, to examine the modified LDPE film’s ability to repel bacterial fouling, an inoculation experiment was conducted using the *E. coli* serotype O157:H7 and staphylococci *S. epidermidis*, employing the agar plate method. The structural features, physical characteristics, and chemical stability of the LDPE film embedded with SRS were examined through a range of methods and techniques, including scanning electron microscopy (SEM), atomic force microscopy (AFM), tensiometry, ultrasonication, mechanical abrasion tests, and Fourier transform infrared (FTIR) spectroscopy.

## 2. Materials and Methods

### 2.1. Preparation of SRS

**Figure 1:**
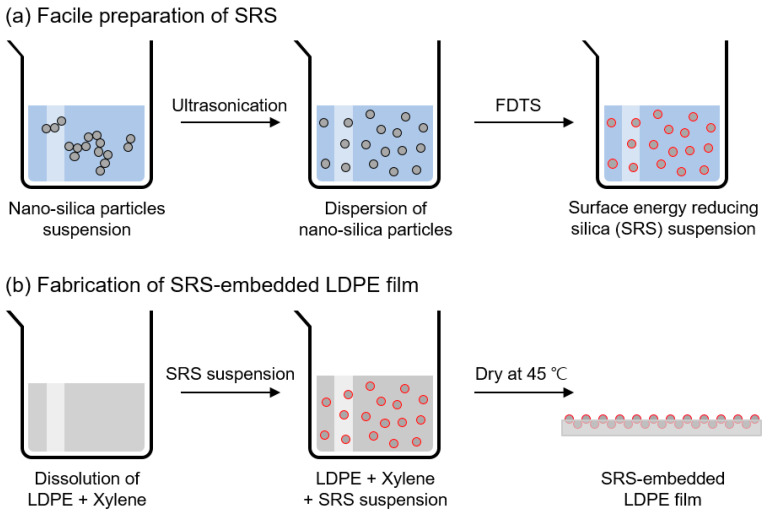
Schematic representation of (**a**) SRS preparation and (**b**) SRS-embedded LDPE film fabrication.

A total of 0.1 g of nonporous nano-silica particles (SiO_2_) with an average size of circa 200 nm, obtained from Sigma-Aldrich (St. Louis, MO, USA), were stably dispersed in 10 mL of nonpolar n-hexane (Daejung Chemicals & Metals, Siheung, Republic of Korea). Subsequently, 35 µL of fluorinated compound trichloro(*1H*,*1H*,*2H*,*2H*-perfluorooctyl)silane (FDTS), which was purchased from Sigma-Aldrich, was introduced to create the surface energy-reducing silica (SRS). The SRS was vigorously suspended via ultrasonication for 30 min using a bath-type ultrasonic processor (WUC-D03H; Daihan Scientific, Ltd., Wonju, Republic of Korea). The resulting SRS suspension was left without disturbance for 1 h to enable a full reaction between the nano-silica particles and FDTS. A schematic illustration of this process is presented in Figure 1a. Upon completion of the reaction, the SRS particles underwent vacuum drying to separate particles from the solvent and were subsequently rinsed three times with hexane. This rinsing procedure was carried out to remove the produced HCl and unreacted FDTS resulting from the chemical grafting reaction between silica particles and FDTS. Following the rinsing steps, the SRS particles were re-suspended in 0.1 g of hexane [36].

### 2.2. Fabrication of SRS-Embedded LDPE Films

Next, 0.15 g of low-density polyethylene (LDPE; Sigma-Aldrich) pellets were readied, rinsed with ethanol (Daejung Chemicals & Metals), and subsequently allowed to air-dry at a room temperature of 21 °C. As shown in Figure 1b, the prepared LDPE pellets were immersed in 10 mL volume of xylene (Daejung Chemicals & Metals). The mixture was stirred until LDPE was completely dissolved in xylene at 130 °C and 200 RPM. For the preparation of bare LDPE film, the solution was poured into a glass dish mold with a diameter of 80 mm, placed on a leveled surface, and then dried under controlled conditions at 45 °C for a duration of 24 h. The process for SRS-embedded LDPE film involves additional steps. Specifically, 1.5 mL of SRS suspension was subsequently added to xylene and stirred vigorously for 5 min at the same temperature. By using the solution-casting method, the mixture of LDPE, xylene, and SRS suspension was transferred to a mold and allowed to dry at 45 °C for a period of 24 h. After the solvent was completely vaporized, the cast SRS-embedded LDPE film was rinsed three times with sterile deionized (DI) water (18.2 MΩ·cm resistivity) to remove impurities and then air-dried at room temperature. The optimized embedding process involved preparing a mixture of LDPE and SRS suspension. This suspension contained modified silicon particles, making up a 1% weight ratio to LDPE. This formulation achieved a contact angle (CA_1.0%_) higher than 160°, representing the optimal contact angle for superhydrophobic surfaces. Test results from different experimental conditions demonstrated the following contact angles: CA_0.5%_ = 127.6 ± 1.7°; CA_1.0%_ = 163.7 ± 2.1°; CA_1.5%_ = 164.1 ± 2.2°; CA_2.0%_ = 163.9 ± 1.3°.

### 2.3. Physical and Chemical Surface Characterization of the LDPE Films

A high-resolution Hitachi S-5200 scanning electron microscope (SEM; Tokyo, Japan) was employed to analyze the surface morphology of the nanotextured LDPE film. In order to improve the clarity and resolution of SEM micrographs, a 7 nm thick platinum layer was applied to the samples before conducting SEM analysis. This was performed to reduce the charging effects on the surfaces of the LDPE film. The SEM was then operated at an emission current of 10 µA and an accelerating voltage of 15 kV.

The nanoscale roughness was analyzed through topological data analysis using Park NX10 atomic force microscopy (AFM; Park Systems, Suwon, Republic of Korea) operating in non-contact mode to prevent any potential damage to the sample surfaces. The roughness average of surfaces (Rq) and root-mean-square roughness (RMS) were analyzed using XEI (Park Systems) software version 1.8.3. The reported roughness values represent the average measurements taken from at least six distinct areas across three separate samples.

Chemical interactions between LDPE and SRS were identified using Fourier transform infrared (FTIR) spectroscopy. The chemical analysis data collected from the FTIR spectrometer (Nicolet iS10, Thermo Fisher Scientific, Waltham, MA, USA) were processed and analyzed with the OMNIC Specta (Thermo Fisher Scientific) software. The wetting properties of the LDPE films were evaluated by measuring the static water contact angles on the LDPE film surfaces using the sessile drop method with a Phoenix 300 (Surface Electro Optics, Siheung, Republic of Korea) contact angle analyzer. Contact angles on bare LDPE film, SRS-coated LDPE film, and SRS-embedded LDPE film were collected by averaging the results of five repetitions, all carried out at room temperature with a consistent water droplet volume of 10 μL. The data were then analyzed with the assistance of a low-bond axisymmetric drop shape analysis plug-in using ImageJ public domain software.

### 2.4. Bacteria Cultivation

For this study, two bacterial strains, namely Gram-negative *Escherichia coli* O157:H7 (ATCC 29522) and Gram-positive *Staphylococcus epidermidis* (ATCC 12228), were obtained from the Korea Culture Center of Microorganisms (Seoul, Republic of Korea). The bacterial cultures were prepared by collecting a loopful (10 µL) of the culture growing on tryptic soy agar (TSA; Becton, Dickinson and Company, Sparks, MD, USA) solid media and transferring it into 9 mL of liquid tryptic soy broth (TSB; Becton, Dickinson and Company) tubes. Subsequently, these cultures were incubated under aerobic conditions at 37 °C without agitation for a duration of 24 h. In the growth medium, the final concentrations of the *E. coli* O157:H7 and *S. epidermidis* suspensions ranged between 8.5 and 9.0 log CFU/mL.

### 2.5. Inoculation of Culture Media on the LDPE Film Surfaces

To inoculate the samples, both the bare and SRS-embedded LDPE films were immersed in 10 mL of bacterial suspensions at room temperature for a duration of 1 h. Afterward, the inoculated samples were carefully taken out from the bacterial suspensions in a single, continuous, and vertical manner. Subsequently, the samples were gently rinsed with 30 mL of DI water to eliminate loosely attached bacteria. Following this, the samples were placed in a sterile dish for further assessments of bacterial adhesion. Any residual bacteria that remained after rinsing with DI water were considered to be attached. All experiments were conducted within a suitable biological safety cabinet using aseptic techniques to maintain sterility and safety. These experiments were repeated five times for accuracy and reliability.

### 2.6. Bacterial Adhesion Assay on the LDPE Film Surfaces

The attachment of bacteria to LDPE film surfaces, both with and without surface modification involving SRS, was evaluated via the plate-counting method for the enumeration of bacteria based on the quantification of colony-forming units per milliliter (CFU/mL). In the plate-counting method, the films were first immersed in the bacterial solution for a period of 1 h. After this, each film was vortexed in 10 mL of 0.1% (*w*/*v*) peptone water for a duration of 10 min to dislodge bacteria from their surfaces. Subsequent to this step, aliquots of 1 mL were taken from the ten-fold serial dilutions prepared from the peptone water containing the detached bacteria and were spread onto TSA plates. The bacterial population densities were then evaluated after 24 h of aerobic incubation, maintaining consistent conditions with the initial bacterial culture incubation. These resulting densities were considered to represent the density of bacteria attached to the LDPE film surfaces. To ensure precise results, all experiments were repeated five times.

### 2.7. Evaluation of Antifouling Properties of the LDPE Film Surfaces against Contaminants

The antifouling characteristics were examined using liquid substances, including sheep blood (Biozoa Biological Supply, Seoul, Republic of Korea), milk, and coffee (*Coffea arabica* extract). Each contaminant was applied onto slightly tilted (i.e., θ = 3.5°) bare and modified films in quantities of 1 mL. After a 10 s interval, the liquid remaining on each surface was observed. Antifouling characteristics against high-viscosity contaminants were tested using ketchup and mustard products from the Kraft Heinz Company (Chicago, IL, USA). Each type of contaminant was placed on both the bare and SRS-embedded LDPE film surfaces, with 1 g of contaminant for each film. Then, 9 mL of DI water was spread onto each film to remove contaminants. Following the water-rinsing treatment, the remaining contaminants on each surface were observed.

### 2.8. Mechanical Durability Test of the LDPE Films

An ultrasonic testing for durability assessment was performed using an ultrasonic processor, operated at 290 W and a frequency of 60 Hz. Both the SRS-coated and SRS-embedded LDPE films were ultrasonicated for 60 min. The presence of surface damage was determined by measuring the static water contact angle, before and after the ultrasonic treatments. The water contact angles of the SRS-embedded LDPE film were compared to those of the SRS-coated LDPE film prepared using a dip-coating technique. The bare LDPE film was divided into square pieces measuring 10 mm × 10 mm. These film pieces were then dipped into the SRS suspension for 1 min and left to air-dry for 1 min, repeating the process five times within a 10 min duration. The SRS-coated films were dried at room temperature for 24 h [37].

To confirm the durability of the superhydrophobic characteristics, an additional wear resistance test was carried out on both the SRS-coated and SRS-embedded LDPE films. A surface-peeling experiment was performed using an abrasion setup. Both the SRS-coated and SRS-embedded LDPE films were exposed to sandpaper (160 grit) attached to a 500 g pendulum counterweight. Both films were maintained in a fixed position during the experiment. The sandpaper was pulled horizontally at a speed of 1 cm/s for 10 s during 50 s. After the test was completed, the remaining superhydrophobicity was evaluated by measuring the static water contact angle.

Furthermore, the chemical stability of the SRS-embedded LDPE film was determined by monitoring chemical leaching from the film submersed in DI water and 2% hydrogen peroxide solution over time. This was accomplished by analyzing the collected aliquots using FTIR spectroscopy, which has a detection limit of less than 1 ppm. The measurements were performed at appropriate submersion durations of 1 day, 1 week, and 2 weeks, which present a sufficient testing conditions.

### 2.9. Statistical Analysis for Analyzing Bacterial Enumeration

Microbiological data were transformed into logarithmic values before conducting statistical analysis. A two-way analysis of variance (ANOVA) with Tukey’s post hoc test was employed to evaluate significant differences in the microbiological data between the bare and SRS-embedded LDPE films, as well as among the different types of bacteria. The significance level was set at *p* < 0.05, and all statistical analyses were carried out using Analysis ToolPak, a statistical package within Microsoft Excel software provided by Microsoft (Redmond, WA, USA).

## 3. Results and Discussion

### 3.1. Characterization of SRS-Embedded LDPE Films

**Figure 2:**
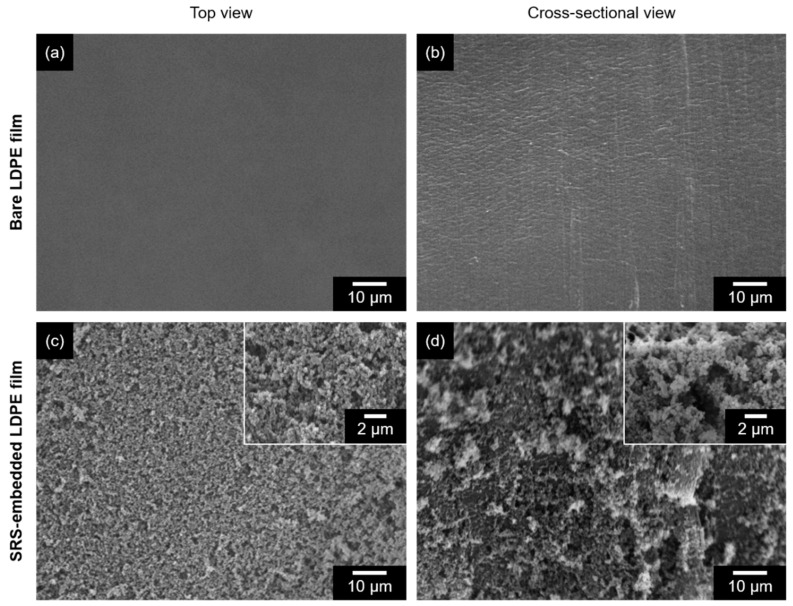
SEM micrographs of (**a**) top view of bare LDPE film, (**b**) cross-sectional view of bare LDPE film, (**c**) top view of SRS-embedded LDPE film, and (**d**) cross-sectional view of SRS-embedded LDPE film. All images were captured at a magnification of ×10,000, and insert images were captured at a magnification of ×30,000.

Figure 2 shows SEM micrographs depicting the surface morphology of the bare and modified LDPE films. Upon examination, the superhydrophobic surfaces exhibited a dense arrangement of spherical-shaped nanostructures, which contributed to the desired surface roughness. Cross-sectional SEM observations provided evidence of nano-silica particles on the surface, confirming the formation of a composite film embedded with SRS, and indicating that the nanoparticles were stably dispersed within the matrix material.

**Figure 3:**
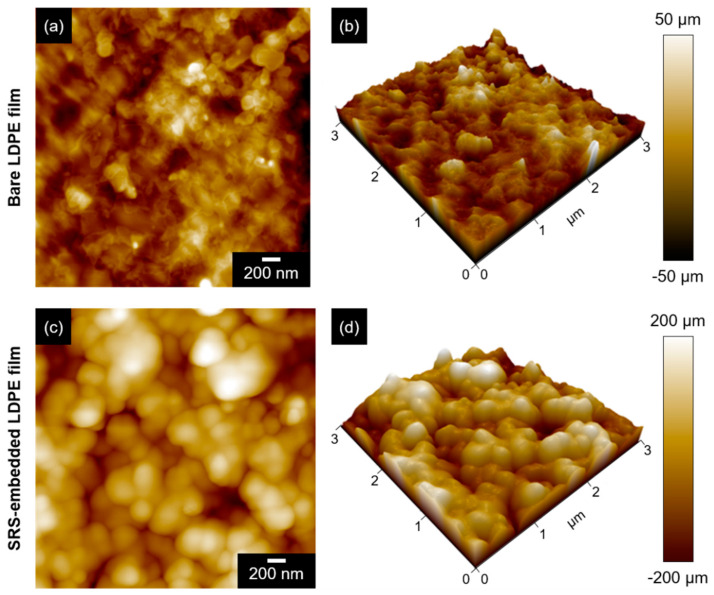
AFM micrographs of (**a**,**b**) bare LDPE films and (**c**,**d**) SRS-embedded LDPE films.

Figure 3 presents AFM data, which were collected to analyze and describe the physical characteristics and surface roughness of the film. The surface topography measurement of the SRS-embedded LDPE film indicates that the root-mean-square roughness (R_q_) was 85.56 ± 2.73 nm, which is smaller than the size of bacteria; for instance, rod-shaped *E. coli* O157:H7 is 1000–2000 nm in length and 500–1000 nm in diameter, and spherical *S. epidermidis* has a diameter of 500–1500 nm. Nanoscale topography was successfully created in the SRS-embedded LDPE film, which prevented the physical attachment and proliferation of bacteria. Studies on the modification of surface roughness at the nanoscale has been shown to prevent the adhesion of bacteria [38]. Surface roughness at a scale larger than the bacterial size provides an increased area for bacterial colonization and residence [39]. Additionally, reducing the contact points through nanoscale roughness decreases the initial adhesion force between bacteria and material surfaces, specifically in biointerfaces [40]. In addition, the roughness features of these coatings should align with the regulations and standards related to surface finishing for food applications. For example, there is a general consensus that surfaces in contact with food should meet specific surface roughness criteria, such as Ra ≤ 0.8 µm, to ensure hygienic equipment design [41].

**Figure 4:**
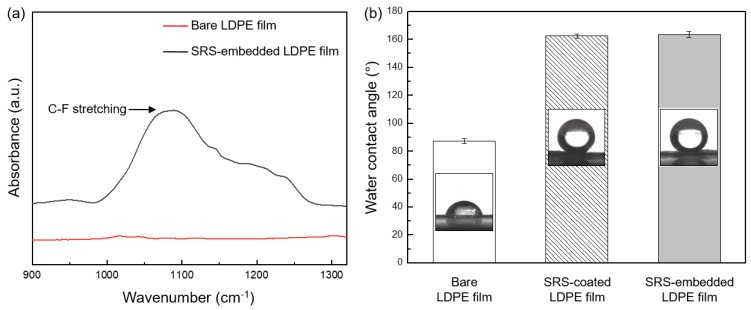
(**a**) FTIR spectra of bare LDPE film (red line) and SRS-embedded LDPE film (black line). (**b**) The water contact angles from the bare LDPE films, SRS-coated LDPE films, and SRS-embedded LDPE films, along with their respective micrographs.

For the chemical modification of the LDPE film, a fluorine-ended silane compound was selected owing to its high reactivity with nano-silica particles [42]. Furthermore, trifluoromethyl (–CF_3_) groups are favored over alternative nonpolar functional groups due to the substitution of hydrogen atoms with fluorine atoms, leading to a reduction in surface energy, with the order being –CF_3_ < –CF_2_H < –CF_2_ < –CH_3_ < –CH_2_ [43]. Surface energy plays a role in bacterial adhesion since bacteria can adhere to surfaces that are both hydrophilic (water-attracting) and hydrophobic (water-repelling). Nonetheless, bacterial adhesion is more frequently observed on hydrophilic surfaces, which generally exhibit higher surface energies [44]. The presence of trifluoromethyl groups on the LDPE film surface was verified via FTIR spectroscopy analysis of the bare and SRS-embedded LDPE film (Figure 4a). A detailed analysis indicated a peak at 1050 cm^−1^, which was assigned to the C–F stretching vibration band of the SRS-embedded LDPE film [45]. The bacterial cell membrane is composed of peptidoglycan, which contains numerous amino acids, *N*-acetylmuramic acid, and *N*-acetylglucosamine [46]. All three of these compounds are functionalized with a hydrophilic terminal group, specifically hydroxy (–OH), carboxyl (–COOH), and amino (–NH_2_) groups [47]. These functional groups result in hydrophilic cell surfaces that deter bacterial adhesion on the superhydrophobically modified SRS-embedded LDPE film surfaces.

Figure 4b shows the static contact angles of water droplets on the bare, SRS-coated, and SRS-embedded LDPE film surfaces. The findings revealed that the bare LDPE film had a nearly hydrophobic angle of θ = 87.5 ± 1.7°. In contrast, the SRS-coated and SRS-embedded LDPE films displayed superhydrophobic angles (θ > 150°), with contact angles of θ = 162.5 ± 1.6° and θ = 163.7 ± 2.1°, respectively. The consistency among these contact angle measurements provides strong evidence for the effective creation of superhydrophobic surfaces achieved through the process of hydrophobization. This accomplishment involved a meticulous procedure that encompassed the deposition of functionalized nano-silica particles and surface modification via chemisorption, resulting in minimal contact angle hysteresis [48].

### 3.2. Bacterial Attachment to SRS-Embedded LDPE Films Characterized via Agar Plate Counting

**Figure 5:**
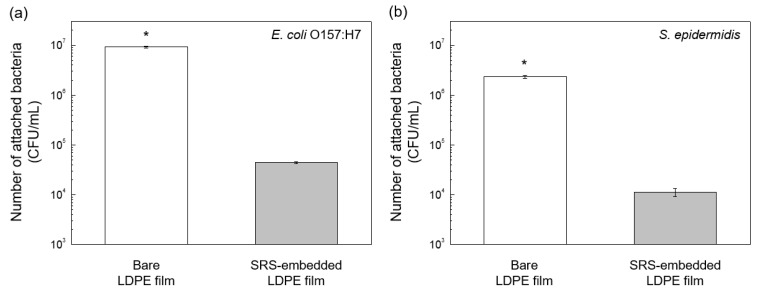
Results of bacterial antifouling tests of bare LDPE films and SRS-embedded LDPE films against (**a**) *E. coli* O157:H7 and (**b**) *S. epidermidis*. Statistical significance is indicated by an asterisk, with *p* < 0.05.

Bacterial growth data on the bare and modified LDPE film surfaces were compared to examine bacterial attachment after modifying the LDPE film surfaces. Figure 5a shows the plate count results of *E. coli* O157:H7 on the bare and SRS-embedded LDPE films after inoculation and attachment. The average bacterial adhesion on the bare LDPE film was determined to be 9.2 × 10^6^ ± 0.4 log CFU/mL, whereas on the SRS-embedded LDPE film, it was 4.4 × 10^4^ ± 0.2 log CFU/mL, representing a 99.5% reduction. The adhesion of *E. coli* O157:H7 between the two surfaces was significantly different (*p* < 0.05). The same bacterial attachment test was conducted again with *S. epidermidis*. Figure 5b shows the plate count results for *S. epidermidis* attached to the bare LDPE and SRS-embedded LDPE films. The average population of *S. epidermidis* attached to the bare LDPE film was 2.3 × 10^6^ ± 0.2 log CFU/mL, whereas on the SRS-embedded LDPE film, it was 1.1 × 10^4^ ± 0.2 log CFU/mL, representing a 99.4% reduction. The adhesion of *S. epidermidis* between the two surfaces was significantly different (*p* < 0.05). The plate count results showed that the adhesion of both *E. coli* O157:H7 and *S. epidermidis* on the SRS-embedded LDPE film decreased by more than 2 log-units when compared to their adhesion on the bare LDPE film. The bacterial antifouling properties of the SRS-embedded LDPE film arises from the synergistic combination of its low surface energy chemistry and nanotextured physical topography, which together create superhydrophobic film surfaces [49]. The majority of bacteria cell walls feature hydrophilic groups on their surface, making it challenging for them to adhere to superhydrophobic surfaces due to the repulsion between the bacterium and the surface. Our modified film incorporates numerous fluorine (–F) groups with low surface energy, significantly reducing the surface energy on the film and effectively resisting bacterial attachment [50]. Additionally, the spherical shape of the silica nanoparticle enhances nanoroughness, inducing air pockets according to the Cassie–Baxter state phenomenon. Bacteria tend to be repelled from these surfaces due to a decrease in real contact area. This results in a weakened interaction between the bacterium and the surface, further contributing to the antifouling properties of the modified film [49].

The plate count results on the same surfaces for the two different bacterial strains were analyzed using two-way ANOVA. The attachments of *E. coli* O157:H7 and *S. epidermidis* to the bare LDPE film surfaces were not significantly different (*p* > 0.05). Similarly, the attachment of these two bacterial strains on the SRS-embedded LDPE film surfaces was not significantly different (*p* > 0.05). This outcome can be explained by the wetting transition from the Wenzel state to the Cassie–Baxter state, which occurs as a result of the surface modification of the LDPE film [51]. The transition to the Cassie–Baxter state signifies the creation of thermodynamically stable air pockets when the bacterial suspensions come into contact with the LDPE film surfaces [52]. This leads to a reduction in the contact area between the LDPE film surfaces and the bacterial culture medium [53]. Our findings can also be interpreted with respect to the hydrophobic effect [54]. Previous research has demonstrated that the angle at which water droplets contacted the bacterial layer collected on a filter varied from 15° to 27° [50]. When these bacteria, which have very hydrophilic cell surfaces, interact with nonpolar surfaces (i.e., fluorinated surfaces), it results in unfavorable intermolecular interactions that prevent bacterial attachment.

### 3.3. Liquid-Repelling Properties of SRS-Embedded LDPE Films

**Figure 6:**
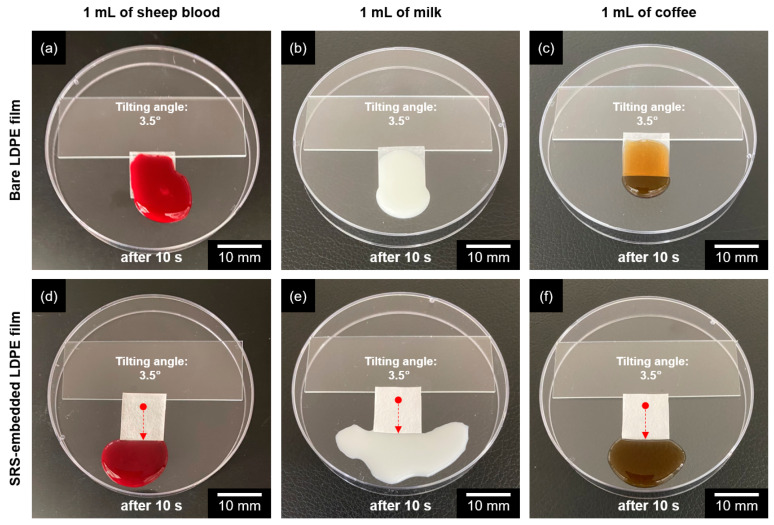
Liquid-repelling properties of (**a**–**c**) bare LDPE films and (**d**–**f**) SRS-embedded LDPE films. The tilting angle of each film was 3.5°. Sheep blood, milk, and coffee were used as food-related contaminants. The red arrows denote the direction of transport for the contaminants.

Enhanced hydrophobicity and roughness are key features of surface-modified LDPE films that prevent the attachment of contaminants to the surface. To investigate the liquid-repelling properties of SRS-embedded LDPE film, sheep blood, milk, and coffee were applied onto the samples placed on the glass slides at a tilting angle of 3.5° (Figure 6). A total of 1 mL each of sheep blood, milk, and coffee was applied onto the bare and modified LDPE films for the comparison. After 10 s, no residual contaminants were attached to the SRS-embedded LDPE film. In contrast, the bare LDPE film exhibited a significant amount of contaminant attachments. Consequently, owing to the superhydrophobic properties of the surfaces, these contaminants can easily roll off without leaving any residue, a feature often referred to as self-cleaning properties [55].

**Figure 7:**
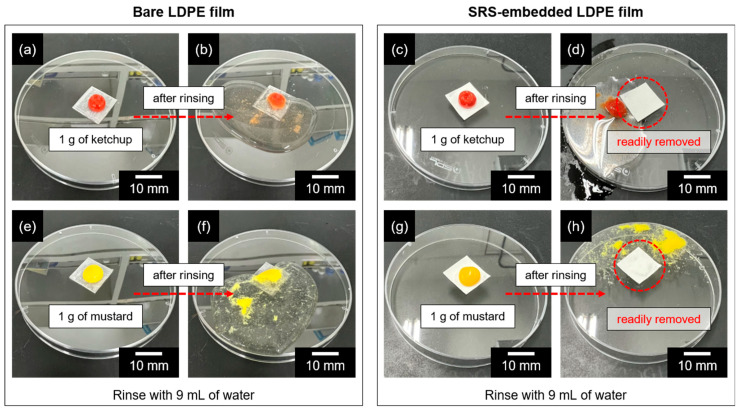
Self-cleaning ability of bare LDPE films and SRS-embedded LDPE films against high-viscosity contaminants, (**a**–**d**) ketchup and (**e**–**h**) mustard.

To confirm its self-cleaning ability, the liquid-repelling properties of the SRS-embedded LDPE film were further examined using ketchup and mustard, which have higher viscosity compared to sheep blood, milk, and coffee (Figure 7). To compare the behaviors, 1 g each of ketchup and mustard was applied to both the bare and SRS-embedded LPDE films. Subsequently, 9 mL of water was spread onto each contaminated film. Although ketchup and mustard adhered to the bare LDPE film, no attachment was observed on the SRS-embedded LDPE film. The surface modification of the LDPE film resulted in complete residue removal after rinsing, indicating a highly effective liquid-repelling and self-cleaning feature compared to the bare LDPE film surfaces.

### 3.4. Mechanical Durability of SRS-Embedded LDPE Films

**Figure 8:**
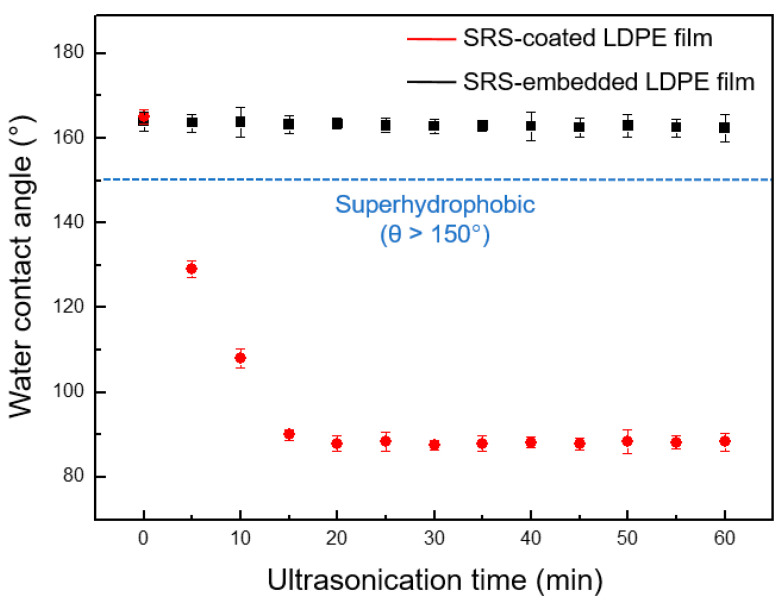
The water contact angles of SRS-coated LDPE films (black line) and SRS-embedded LDPE films (red line) measured under ultrasonication for up to 60 min.

To further investigate the mechanical durability of the modified film, specifically its superhydrophobic properties, ultrasonic dynamic mechanical tests were conducted. A comparison was made between LDPE films embedded with SRS and films coated with SRS using a dip-coating method [56], which is one of the most commonly used coating techniques, in order to evaluate their mechanical durability. Ultrasonic testing was conducted with a bath-type ultrasonic processor, and the static contact angle was monitored for a duration of up to 60 min using a contact angle analyzer. As shown in Figure 8, the SRS-embedded LDPE film retained its superhydrophobic properties for 60 min. The water contact angle of the SRS-embedded LDPE film after ultrasonic testing was measured at 163.2 ± 2.3°. In contrast, the dip-coated SRS nanoparticles in the LDPE film were peeled off by the ultrasonic testing process. The water contact angle drastically decreased over the course of 15 min within the 1 h period. The final water contact angle of the LDPE film dip-coated with SRS was 88.3 ± 2.7°, which is similar to that of the bare LDPE film. These results indicated that the SRS-embedded LDPE film exhibited greater mechanical durability to ultrasonic treatment compared to the SRS-coated LPDE film. Moreover, the durability was validated through surface water flow measurements, simulating dynamic conditions similar to washing and rinsing practices (please see Figure S1 in the Supplementary Materials).

**Figure 9:**
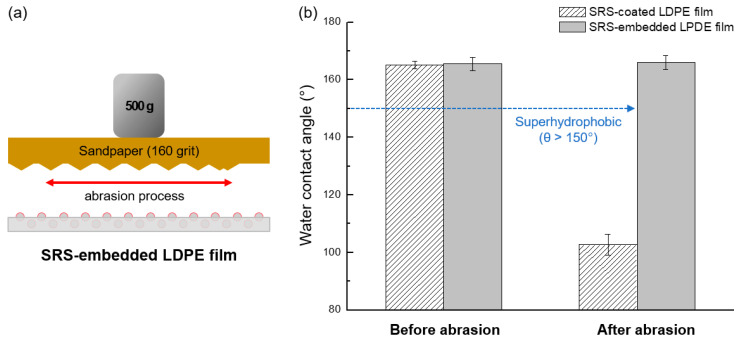
(**a**) Schematic illustration of surface abrasion resistance test conducted using sandpaper, and (**b**) the water contact angles of SRS-coated LDPE films and SRS-embedded LDPE films before and after abrasion.

To assess the mechanical durability and ability to maintain superhydrophobic characteristics, a surface scratch abrasion test was conducted. The SRS-coated and SRS-embedded LDPE films were exposed to sandpaper with a 500 g pendulum counterweight applied on top (Figure 9). The sandpaper was horizontally moved at a speed of 1 cm/s for a total duration of 10 s under fixed conditions. The water contact angles of the LDPE films were measured before and after the test to evaluate the changes in their surface properties.

**Figure 10:**
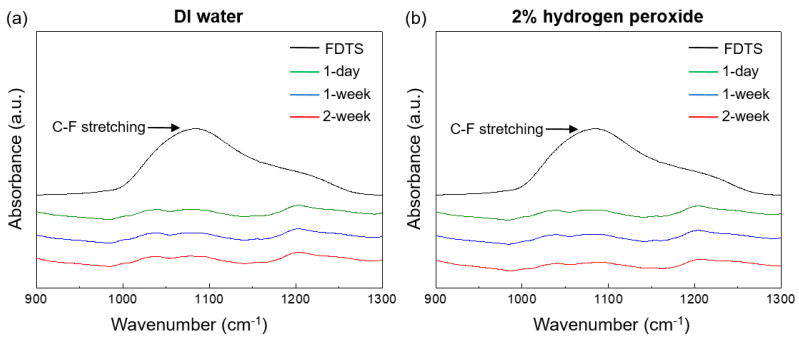
Chemical leaching test in (**a**) DI water and (**b**) 2% hydrogen peroxide solution for durations of 1 day (red line), 1 week (blue line), and 2 weeks (green line) with a detection limit as low as 1 ppm. The black line represents the silane compound terminated with fluorine.

The potential toxicity of the modified film is associated with its ability to leach chemicals from SRS nanoparticles via the degradation, decomposition, or detachment of fluoro groups [57]. The chemical stability of the SRS-embedded LDPE film in DI water and 2% hydrogen peroxide solution over time, obtained via FTIR spectroscopy, is shown in Figure 10. The FDTS molecules that were not bound displayed asymmetric and symmetric C–F stretching vibrations around the 1000–1050 cm^−1^ region. The spectroscopic analysis of the SRS-embedded LDPE film submersed in both DI water and 2% hydrogen peroxide solution revealed that there was no chemical leaching. The analysis revealed that there were no detectable free chemicals within the limit of detection, which was set at 1 ppm, during the 1-day, 1-week, and 2-week observation periods.

## 4. Conclusions

This study focuses on the surface modification of LDPE films with surface energy-reducing silica (i.e., SRS) to prevent bacterial attachment. The bacterial antifouling and liquid-repelling properties of SRS-embedded LDPE film were investigated using bacteria associated with foodborne illness and various food-related contaminants. The SRS-embedded LDPE film showed a superhydrophobic property, with a contact angle (water) higher than 160° and low sliding angle (3.5°), indicating sufficiently low surface energy to prevent biological and non-biological contaminants. The plate-counting method after dip-inoculation showed substantially greater bacterial attachment to the bare LDPE film than to the superhydrophobically modified LDPE film, with a reduction of over 2 log-units. This superhydrophobicity minimizes the contact between the modified film surfaces and contaminants, thus decreasing the probability of bacteria and contaminants with both low and high viscosity adhering to the LDPE film surfaces. Also, the SRS-embedded LDPE film demonstrated excellent durability, mechanical robustness, and chemical stability. Overall, the bacterial antifouling and liquid-repelling properties of the SRS-embedded LDPE film present a promising solution for enhancing food hygiene and ensuring microbiological food safety in the food industry.

## Data Availability

Data are contained within the article and supplementary materials.

## Supplementary Materials

The following supporting information can be downloaded at: https://www.mdpi.com/article/10.3390/polym16020292/s1, Figure S1. The water contact angles of SRS-embedded LDPE films were assessed following surface water flow measurements under flow conditions for a duration of up to 144 h.
