# Supplementary material for: Durable Surface Modification of Low-Density Polyethylene/Nano-Silica Composite Films with Bacterial Antifouling and Liquid-Repelling Properties for Food Hygiene and Safety
Source: Polymers (Basel). 2024 Jan 21;16(2):292. doi: 10.3390/polym16020292 (PMC10819097; doi:10.3390/polym16020292)
Supplement: Supplementary file 1 [file polymers-16-00292-s001.zip › polymers-2753520-supplementary.pdf]

Durable surface modification of low-density polyethylene/nano-silica composite films with bacterial antifouling and liquid-repelling properties for food hygiene and safety

Sang Ha Song[1], Michael Bae[2,*], Jun Kyun Oh[1,*]

[1]*Department of Polymer Science and Engineering, Dankook University, 152 Jukjeon-ro, Suji-gu, Yongin-si, Gyeonggi-do 16890, Republic of Korea*

[2]*Artie McFerrin Department of Chemical Engineering, Texas A&M University, College Station, TX 77845, USA*

*Correspondence and requests for materials should be addressed to M. Bae (email: bsy7790@tamu.edu) or J.K. Oh (email: junkyunoh@dankook.ac.kr).

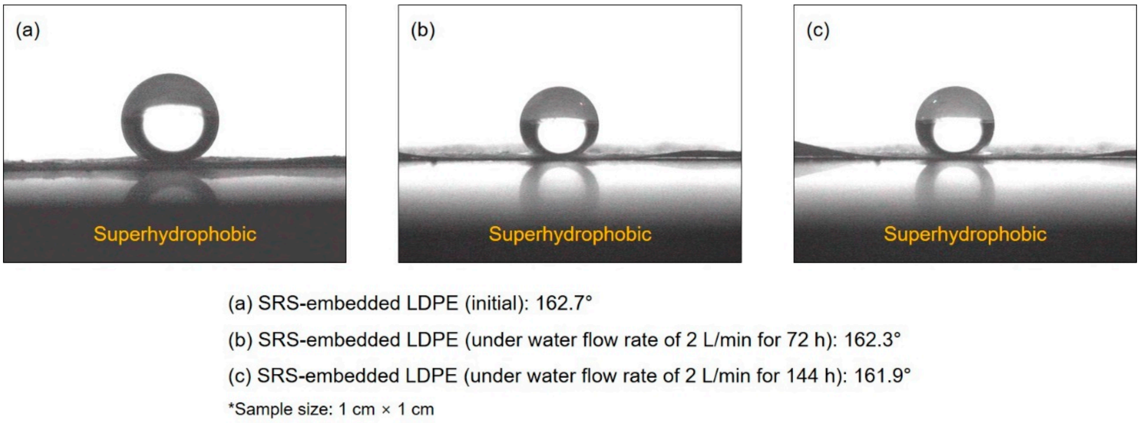

Figure S1. The water contact angles of SRS-embedded LDPE films were assessed following surface water flow measurements under flow conditions for a duration of up to 144 h**.**
